# Ultrashort-Segment Hirschsprung's Disease Complicated by Megarectum and Obstructive Uropathy: A Case Report

**DOI:** 10.7759/cureus.48851

**Published:** 2023-11-15

**Authors:** Wyatt R Glasgow, Dimitrios Lintzeris, Leon Stockton, Zvi Harris

**Affiliations:** 1 General Surgery, Campbell University School of Osteopathic Medicine, Lillington, USA; 2 General Surgery, University of North Carolina, Chapel Hill, USA; 3 Wound Care, University of North Carolina, Chapel Hill, USA

**Keywords:** adult hirschsprung's disease, acute large bowel obstruction, emergency exploratory laparotomy, congenital aganglionic megacolon, hirschsprung's disease

## Abstract

Adult Hirschsprung's disease (AHD) is a rare condition characterized by a shortened aganglionic segment in the distal colon or rectum that is diagnosed after the age of 10. Diagnostic challenges stem from its rarity, nonspecific presentation, and often delayed consideration following emergent interventions. This report details the case of a 33-year-old male who presented with chronic constipation and abdominal pain, leading to a severe bowel obstruction attributed to self-reported Hirschsprung's disease (HD). Clinical, radiological, and historical aspects were suggestive of AHD, but definitive diagnostic procedures, including manometry and biopsy, were hindered by the patient's deteriorating condition. Exploratory laparotomy unveiled a secondary small bowel obstruction due to volvulus, necessitating immediate intervention, resulting in the removal of 4000 cc of fecal material. A comprehensive resection involving mid-to-distal transverse colon, left colon, sigmoid colon, and proximal rectum with the creation of Hartman's colostomy was performed due to the patient's worsening clinical status. We present a case of possible ultrashort-segment Hirschsprung's disease (USHD) and sketch a classic presentation of AHD. This endeavor aims to enhance awareness and consideration of AHD and/or USHD within the spectrum of potential diagnoses for chronic constipation when relevant and demonstrate the effectiveness of surgical intervention in this population.

## Introduction

Although Hirschsprung's disease (HD) is generally recognized as a congenital condition diagnosed in neonates, specific situations have led to its underdiagnosis. Adult Hirschsprung's disease (AHD) presents as a diagnostic challenge due to its rarity and presentation following emergency interventions.

In normal development, embryonic neural crest cells migrate along the vagus nerves in a craniocaudal direction concurrently with the development of the intestinal tract [1]. These cells respond to intricate environmental factors during migration, and the imbalance of these cues, such as the absence or overabundance of certain factors (e.g., laminin), has been hypothesized to contribute to HD. These findings suggest that imbalance of signaling factors leads to segments of the myenteric plexus lacking ganglia, thereby impeding proper relaxation and peristalsis due to, for example, the overexpression of laminin [2] or the underexpression of serotonin [3-4].

The estimated prevalence of the disease is around one in 5000 live births, with a male-to-female ratio of 4:1 [5-6], which may vary among ethnic groups. Approximately half of individuals with HD receive their diagnosis in the first year of life [7-8], 80% are diagnosed by age 7, and over 90% receive a diagnosis by age 13 [7-8]. Of those, 80% manifest as a short-segment condition, with the aganglionic bowel segment limited to the rectosigmoid area [9]. However, reports of AHD have been on the rise. These patients are diagnosed after the age of 10 and rarely present with classic symptoms such as tight anal sphincter or failure to pass meconium. These patients, typically young men, often suffer from a history of chronic, refractory constipation and abdominal distention managed with laxatives, enemas, and occasionally manual de-impaction [9]. Upon presentation, this history of progressive dilation of the upstream colon shows very characteristic radiologic findings [10]. Interestingly, sigmoid volvulus (SV), as seen in our patient, is a rare complication of AHD, with a reported prevalence of 0.66% [11]. It should be noted that in younger children (<14) presenting with SV, prevalence of HD increases to 18% [12]. 

A further variant of HD, ultrashort-segment Hirschsprung's disease (USHD), may also be to blame [13]. While demonstrating all the same presentations of AHD, USHD presents as an aganglionic rectal segment >1-2 cm in the distal rectum or colon [14], alluding biopsies or barium enemas for definitive diagnosis.

## Case presentation

A 33-year-old chronically ill-appearing white male presented to the emergency department (ED) via emergency medical services (EMS) from his home with complaints of worsening abdominal pain, distention, and vomiting. The patient had a past medical history notable for HD, which he stated had been diagnosed during his childhood; however, no records were found to confirm this. He had not undergone any previous abdominal surgeries related to this condition. The patient reported a chronic pattern of infrequent bowel movements, typically occurring every five days and necessitating the use of laxatives for relief. However, his current presentation was marked by an exacerbation of his chronic constipation, leading to an alarming array of symptoms over the past 10 days. The patient's primary complaints included worsening abdominal pain, abdominal distention, nausea, vomiting, and an inability to pass stool. His abdominal pain was described as severe, cramping, intermittent, and non-radiating, with a numeric rating of 10/10. Additionally, he reported decreased urine output and difficulty urinating, accompanied by a suspicion of stool in his urine. Notably, the patient denied any symptoms suggestive of shortness of breath, even though his physical appearance displayed tachypnea and diaphoresis.

On physical examination, the patient's appearance was thin and older than his stated age, consistent with chronic illness. Vital signs upon arrival were stable: blood pressure 104/64 mm Hg, pulse 65 beats per minute, temperature 36.6°C (97.8°F), respiratory rate 17 breaths per minute, weight 68 kg (150 lb), oxygen saturation 99% on room air, and BMI 18.75 kg/m². A morphine intravenous (IV) injection of 4 mg was administered to alleviate pain, and a 1 L bolus of lactated Ringer's solution was given for hydration. Given the severity of the patient's symptoms and his history of HD, a comprehensive work-up was initiated. Laboratory studies revealed a significant leukocytosis, with a white blood cell count of 29.1, characterized by neutrophilic predominance. A comprehensive metabolic panel showed notable findings, including hyponatremia (sodium 135 mmol/L), mildly elevated blood urea nitrogen (BUN) (14 mg/dL), mildly decreased creatinine (0.50 mg/dL), and hyperglycemia (glucose 174 mg/dL). Notably, the lactic acid level was within normal limits at 1.5 mmol/L, and the lipase level was unremarkable at 23 U/L. Viral swab results were unremarkable. In light of these findings, the patient was initiated on IV Zosyn (Baxter International, Deerfield, Illinois, United States) for broad-spectrum antimicrobial coverage.

**Figure 1:**
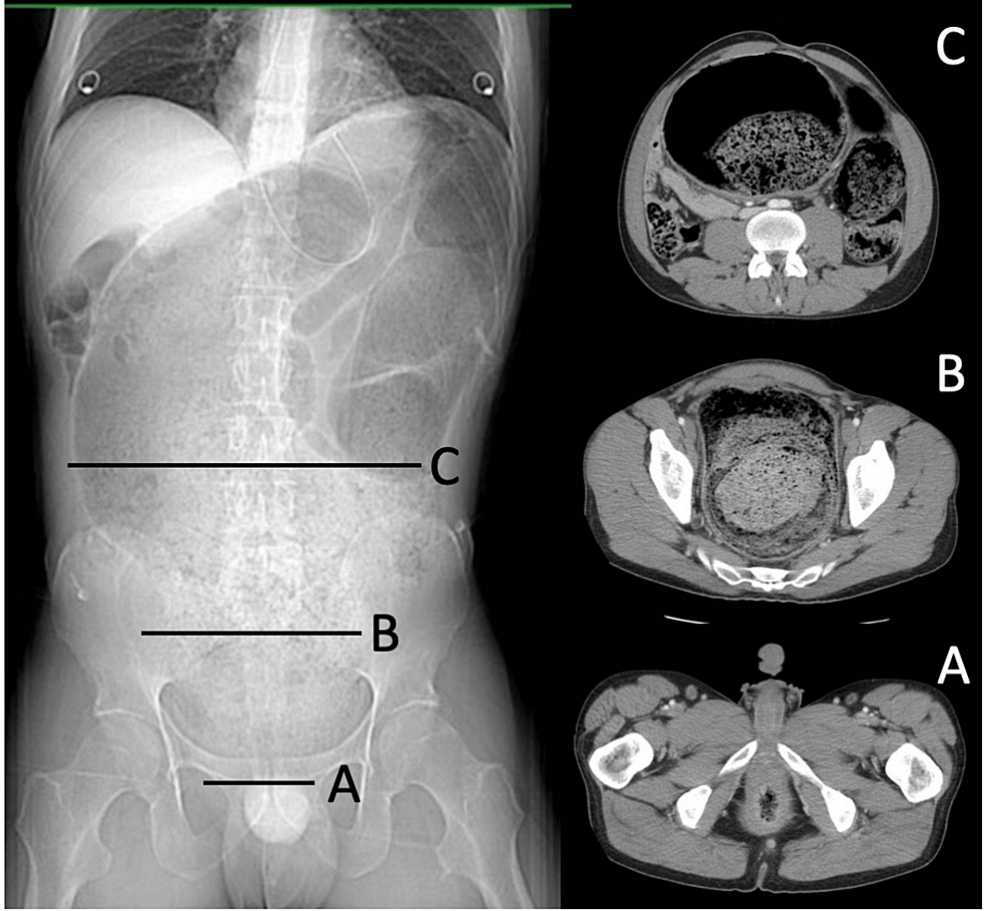
CT scan of the abdomen/pelvis with contrast. A. Anus. B. Transition point: dilated rectum with stool; bladder compressed right and anteriorly. C. Redundant L colon/R colon with obstruction. CT: computed tomography; L: left; R: right

**Figure 2:**
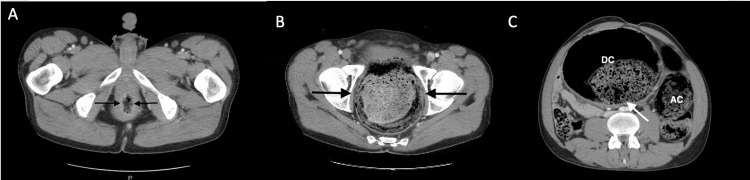
AHD with the aganglionic segment of the lower part of the rectum in a 33-year-old man. (a,b) Contrast-enhanced transverse CT scans show markedly dilated feces: filled proximal upper part of the rectum and the rectosigmoid junction (arrows in b) with a transition zone and a distal narrowed rectum (arrows in a). The distal rectum also appears to also have a thickened wall. (c) Contrast-enhanced transverse CT scans (b obtained at lower level than c) obtained in a 33-year-old man with AHD show markedly dilated feces: filled AC and DC with sigmoid volvulus. AHD: adult Hirschsprung's disease; CT: computed tomography; AC: ascending colon; DC: descending colon

Computed tomography (CT) imaging of the abdomen and pelvis was performed and demonstrated an alarming picture. The colon (Figures 1C, 2C), sigmoid (Figures 1B, 2B), and rectum (Figures 1A, 2A) exhibited marked dilatation, with an extensive impaction of stool. This impaction not only was evident in the rectal vault but also extended into the left colon. Notably, the bladder was anteriorly and laterally displaced due to the distended rectum, leading to mild left hydronephrosis and trace left hydroureter. The patient was promptly assessed by a surgical consultant, who confirmed the need for immediate intervention (Figure 1, Figure 2).

In the ED, the patient's management plan was meticulously outlined. He was administered a milk of magnesia enema, albeit with limited success. General surgery was consulted, and the recommendation was made to initiate Golytely (Braintree Laboratories, Inc., Braintree, Massachusetts, United States), a polyethylene glycol-based cathartic, at a continuous rate of 100 ml/hr overnight. A Foley catheter was inserted to address his urinary symptoms, and antiemetic therapy with Zofran (GSK plc, London, England, United Kingdom) was prescribed to alleviate nausea. IV morphine was made available for pain management. The patient's diet was restricted to nil per os/nothing by mouth (NPO), with medications administered via tube.

Upon admission to the hospitalist service, the patient was administered 10 mg of Reglan (ANI Pharmaceuticals, Inc., Baudette, Minnesota, United States) to alleviate nausea and stimulate gastrointestinal (GI) motility. Two doses of 100 ml of Golytely were administered via a gastrostomy tube, along with attempts at a milk and molasses enema, all of which yielded limited success. The Foley catheter remained in place, with the urine exhibiting a concerning brown coloration. The patient continued to receive IV fluids and Zosyn for antimicrobial coverage. Despite these efforts, the patient's symptoms persisted, and his condition necessitated further escalation of treatment.

The following morning, nursing documentation indicated the patient experienced two episodes of brown-colored emesis with the abdomen remaining firm and distended overnight. The decision was made to connect the nasogastric tube (NGT) to low intermittent suction due to the patient's inability to tolerate the Golytely regimen. At this juncture, the patient had completed approximately half of the Golytely container.

Subsequently, at approximately 10:30 am, the patient was evaluated by general surgery. Physical exam indicated a very tight and grossly distended abdomen with high-pitched bowel sounds. Despite diffuse abdominal tenderness to palpation, there was no guarding or rebound. However, the abdomen exhibited diffuse rigidity. Notably, feculent material was noted in the NGT, and dark blood-colored urine was observed in the Foley bag. At this time, the patient remained hemodynamically stable and afebrile, but remained tachycardic with worsening tachypnea. When speaking with the patient, it was explained that optimally, the treatment for this disease would undergo bowel prep and then a surgical resection of the rectum and sigmoid colon followed by a pull-through procedure with diverting loop colostomy. However, given the patient's deteriorating clinical state and rigid abdomen, the decision was made to take the patient for an emergency exploratory laparotomy due to concerns of impending perforation.

Surgical intervention and intraoperative findings

The patient's rectum and sigmoid colon were massively dilated, and a rock-hard fecal impaction measuring approximately 12 cm in diameter was identified within the rectum. The distal left colon and transverse colon were also dilated, albeit to a lesser extent. The SV was addressed by untwisting the sigmoid colon, and meticulous mobilization of the colon was undertaken. This included mobilization of the proximal transverse colon and ligation of the splenocolic and gastrocolic ligaments. The staple line on the proximal transverse colon was open. The fecal material (over 4000 cc) within the remainder of the colon was then milked backward through the staple line into a stainless steel tunnel. The mid-transverse colon was transected, and attention was shifted toward the rectum.

**Figure 3:**
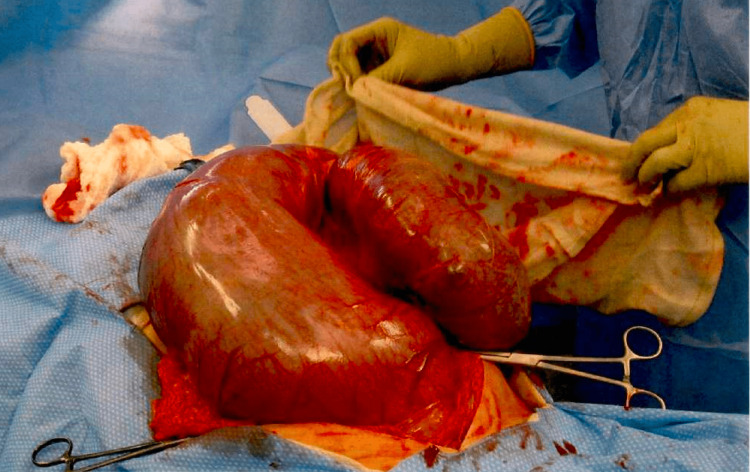
Surgical specimen: sigmoid colon and rectum. Post resection with volvulus corrected anatomically.

**Figure 4:**
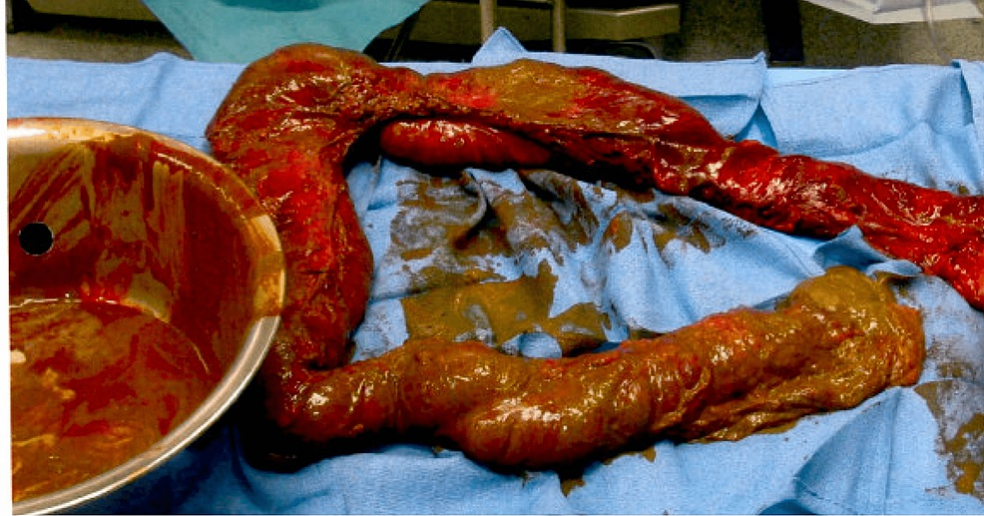
Surgical specimen: mid-transverse colon and L colon. Post resection with stool removed. L: left

The rectum was mobilized and meticulously dissected, revealing the massive fecal impaction approximately 12 cm in diameter. It was resistant to manual manipulation and required subsequent disimpaction with a ring forceps, during which multiple pieces of the impacted stool were removed. Subsequent steps included removal of the distal rectum and sigmoid colon and the creation of a Hartman's colostomy to address the severe obstruction. The surgery concluded with thorough irrigation, wound closure, and placement of a wound vacuum-assisted closure (VAC) to promote healing. See Figure 3 and Figure 4 showing the intraoperative surgical specimens.

Postoperative course and outcome

Following the surgery, the patient was transferred to the intensive care unit (ICU) for close monitoring and management. He initially remained intubated and required mechanical ventilation due to respiratory failure but was gradually weaned off ventilatory support and successfully extubated. His postoperative course was marked by challenges, including electrolyte abnormalities, urinary tract infections (UTIs), and wound care management.

The patient's condition steadily improved over the subsequent days, with significant reductions in leukocytosis and improvements in urinary output and renal function. He was eventually transitioned to room air, underwent successful colostomy care education, and demonstrated independence in managing his colostomy. With family support and adequate wound healing, the patient's antibiotics were completed, and he was discharged with a wound VAC and home health support.

Pathology results reported a bowel specimen 110 cm in length, 9.2 cm proximally and 16 cm distally at the sigmoid/rectum. The distal end of the bowel wall is slightly thickened and has a rubbery consistency, with a thickness of 0.6 cm. Interestingly, histologic examinations conducted every 10 cm showed submucosal and myenteric ganglion cells in all sections.

## Discussion

In Miyamoto's comprehensive review [15], he presents a detailed analysis and typical presentation for AHD, which has since been expanded upon by others [9,16-17]. The review outlines a predominant male population with an average age of 24 years, highlighting their chronic constipation since early life, manifesting with intervals of one week to two months between bowel movements. As they mature, a substantial portion (73-92%) attempt self-remedies. Eventually, as exemplified in our case, some patients experience aggravated constipation culminating in a full obstruction. Consequently, patients report characteristic symptoms including abdominal distension (more than 90% cases), vomiting (more than 85% cases) which may be bilious [16], and pain and tenderness (40-80% of cases) [15]. Notably, more than 50% of cases involve palpable fecal matter. Unidentified HD can manifest as colonic volvulus, resembling the clinical presentation of functional or obstructive megacolon in adults, and has been associated with significant GI and respiratory complications [17]. 

When clinical suspicion arises, the diagnostic work-up for HD typically encompasses a series of investigative measures, including imaging studies, anal manometry, and full-thickness rectal biopsies [18]. Biopsies typically show HD aganglionosis localized to the rectum (79.8%); however, it can also be present in the rectosigmoid junction (12.5%) and, less commonly, the descending colon (0.8%) [19]. An indicative presentation of AHD often includes a significantly dilated proximal colonic segment displaying a transition zone with a constricted distal colonic segment on CT scans and double-contrast barium enemas [19]. The term "transition zone" is used to refer to a portion of the colon where a well-innervated intestine widens as it descends into the aganglionic segment, which narrows down [20]. Traditionally, a barium enema would typically show a constricted distal colon with enlargement upstream, which is a classic sign. Nevertheless, this characteristic observation might not be apparent in adults when the condition is confined to the external anal sphincter. 

In adults, HD rarely presents as acute surgical emergencies like toxic megacolon or intestinal obstruction, and in such cases, SV is associated with an increased mortality rate [17]. In this patient with rapidly declining status and concern for perforation, the typical diagnostic work-up could not be completed; however, it was included here for completeness. It should be noted that while this case presents the short-segment variant of AHD, many other subtypes have been identified including intestinal neuronal dysplasia, isolated hypoganglionosis, absence of the argyrophil plexus, and internal anal sphincter achalasia, among others [14,21-23]. 

The definitive intervention for any HD involves surgical measures aimed at removing any gangrenous bowel segments, excision of aganglionic segments (in this case, the rectum), and establishing bowel continuity between normally innervated bowel and anal canal to restore long-term continence. Surgical options include the following: Duhamel's procedure employs a retro-rectal transanal pull-through without necessitating rectal transection. Soave's procedure, another avenue, entails rectal mucosal stripping while preserving the muscular cuff, followed by the introduction of a ganglionic colon segment and subsequent coloanal anastomosis. An alternative approach, Swenson's procedure, integrates a leveling strategy incorporating additional mucosal biopsies along the antimesenteric border of the sigmoid to determine the presence of ganglionated bowel. This technique involves rectal mobilization and excision of the affected bowel and rectum and culminates in a coloanal anastomosis. Previously, Duhamel's procedure has been reported as the most successful and with the least number of complications [24]; however, newer analysis indicates Soave's and Duhamel's procedures have the least number of complications [25-26] and Swenson's procedure has the most among the three [9]. It is important to highlight that a protective colostomy has been reported to positively affect the outcomes of the procedure [8-9,15-17]. In our patient's case, the large bowel from the sigmoid to the mid-transverse colon exhibited widespread colonic dilatation, ectasia, and dusky tissue, which necessitated a subtotal colectomy. 

## Conclusions

This case report highlights the intricate management of a complex presentation of severe fecal impaction and colonic obstruction in an adult patient with HD. Timely surgical intervention, multidisciplinary collaboration, and meticulous postoperative care were instrumental in the patient's successful recovery and discharge. While AHD remains a rare entity, this case underscores the importance of recognizing and addressing this condition promptly to mitigate potential life-threatening complications. It also underscores the vital role of interdisciplinary communication and the challenges involved in managing complex GI conditions in a clinical setting.
